# RAACBook: a web server of reduced amino acid alphabet for sequence-dependent inference by using Chou’s five-step rule

**DOI:** 10.1093/database/baz131

**Published:** 2019-12-05

**Authors:** Lei Zheng, Shenghui Huang, Nengjiang Mu, Haoyue Zhang, Jiayu Zhang, Yu Chang, Lei Yang, Yongchun Zuo

**Affiliations:** 1 State Key Laboratory of Reproductive Regulation and Breeding of Grassland Livestock, College of Life Sciences, Inner Mongolia University, Zhaojun Road No.24, Hohhot, 010070, China; 2 College of Bioinformatics Science and Technology, Harbin Medical University, Baojian Road No.157, Harbin 150081, China

## Abstract

By reducing amino acid alphabet, the protein complexity can be significantly simplified, which could improve computational efficiency, decrease information redundancy and reduce chance of overfitting. Although some reduced alphabets have been proposed, different classification rules could produce distinctive results for protein sequence analysis. Thus, it is urgent to construct a systematical frame for reduced alphabets. In this work, we constructed a comprehensive web server called RAACBook for protein sequence analysis and machine learning application by integrating reduction alphabets. The web server contains three parts: (i) 74 types of reduced amino acid alphabet were manually extracted to generate 673 reduced amino acid clusters (RAACs) for dealing with unique protein problems. It is easy for users to select desired RAACs from a multilayer browser tool. (ii) An online tool was developed to analyze primary sequence of protein. The tool could produce K-tuple reduced amino acid composition by defining three correlation parameters (K-tuple, g-gap, λ-correlation). The results are visualized as sequence alignment, mergence of RAA composition, feature distribution and logo of reduced sequence. (iii) The machine learning server is provided to train the model of protein classification based on K-tuple RAAC. The optimal model could be selected according to the evaluation indexes (ROC, AUC, MCC, etc.). In conclusion, RAACBook presents a powerful and user-friendly service in protein sequence analysis and computational proteomics. RAACBook can be freely available at http://bioinfor.imu.edu.cn/raacbook.

Database URL: http://bioinfor.imu.edu.cn/raacbook

## Introduction

With the development of various biotechnologies, the number of protein sequences is growing at a rapid pace. However, the three-dimensional structures and function of most proteins are still not determined. For example, in August 2019, there are 154 939 structures, 560 537 reviewed proteins and 167 761 270 unreviewed protein sequences in Protein Data Bank (PDB) (1, 2), the Swiss-Prot and TrEMBL (3), respectively. Obviously, the gaps between structure data, function data and protein sequences are increasing fast. Although X-ray crystallography is a powerful tool in determining these structures, it is time-consuming and expensive, and not all proteins can be successfully crystallized. Membrane proteins are difficult to crystallize, and most of them will not dissolve in normal solvents. Therefore, so far few membrane protein structures have been determined. NMR is indeed a very powerful tool in determining the 3D structures of membrane proteins (4–21), but it is also time-consuming and costly. Thus, it is urgent to design efficient computational methods based on sequence information for rapidly and accurately identifying biological features in primary protein sequences.

Subsequently, experimental interest in reduced alphabet was firstly proposed in the 1960s (22). Alphabet reduction techniques play high-potential roles for sequence alignment and topological estimation (23), which have been widely used in almost all of protein classification (24–32). Meanwhile, a series of 3D protein structures have been developed by means of structural bioinformatics tools (33–45). Facing the explosive growth of biological sequences discovered in the postgenomic age, to timely use them for drug development, a lot of important sequence-based information, such as PTM (post-translational modification) sites in proteins (46–87), protein–drug interaction in cellular networking (88), protein–protein interactions (89), DNA-methylation sites (90), recombination spots (91) and sigma-54 promoters (92), have been deducted by various sequential bioinformatics tools such as the PseAAC approach and PseKNC approach (93). Recently, success of AlphaFold on creating 3D protein models proved that the sequence-dependent inference has incredible potential in computational proteomics (94). Actually, rapid development in sequential bioinformatics and structural bioinformatics has driven the medicinal chemistry undergoing an unprecedented revolution (54), in which the computational biology has played increasingly important roles in stimulating the development of finding novel drugs (95, 96).

**Figure 1:**
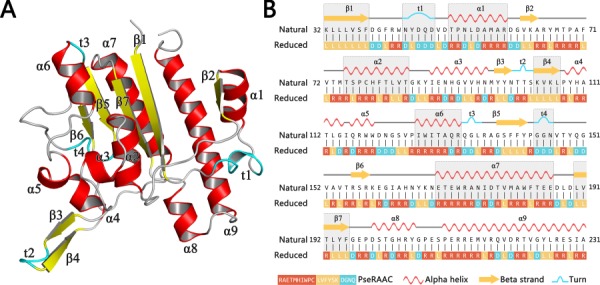
A schematic view of a protein 5TCD in PDB with secondary structures. Subfigure (**A**) shows the three-dimensional structure of this protein. All secondary structural elements are indicated as different labels. Subfigure (**B**) shows its corresponding chain view, where the gray background represents the portion of the reduced amino acid sequence that matches the protein secondary structural elements.

By clustering around 20 amino acids to smaller alphabet based on some similar rules, the protein complexity will be dramatically reduced, and some functional conserved regions will be more clearly displayed (97). For example, Figure 1A shows a schematic view of a protein 5TCD, which is ectonucleotide pyrophosphatase. Its decreased levels may be involved in colon cancer. By utilizing the analysis of amino acid reduction, we can clearly find the correlation between the primary sequence and its 3D structure (Figure 1B). The unique sequence bias of the three-dimensional structure can be visualized in a one-dimensional interface, which shows that reduced amino acid clusters (RAACs) have sufficient capability to identify the consensus domain in sequence alignment (98). Recent work demonstrated that the specific codes endow sequence motifs with unique structures or functions, and the differential combination and arrangement of the motifs with specific codes determine the protein isoforms that possesses multiple functions (99).

With the explosive growth of biological sequences in the postgenomic era, one of the most important but also most difficult problems in computational biology is how to express a biological sequence with a discrete model or a vector, yet still keep considerable sequence-order information or key pattern characteristic. This is because all the existing machine-learning algorithms (such as ‘Optimization’ algorithm (100), ‘Covariance Discriminant’ or ‘CD’ algorithm (101, 102), ‘Nearest Neighbor’ or ‘NN’ algorithm (103) and ‘Support Vector Machine’ or ‘SVM’ algorithm (103, 104)) can only handle vectors as elaborated in a comprehensive review (105).

However, a vector defined in a discrete model may completely lose all the sequence-pattern information. To avoid completely losing the sequence-pattern information for proteins, the pseudo amino acid composition (93) or PseAAC (106) was proposed. Ever since the concept of Chou’s PseAAC was proposed, it has been extensively used in nearly all the areas of computational proteomics (107–116). Because it has been widely and increasingly used, four powerful open-access softwares, called ‘PseAAC’ (117), ‘PseAAC-Builder’ (118), ‘propy’ (119) and ‘PseAAC-General’ (120), were established: the former three are for generating various modes of Chou’s special PseAAC (121), the fourth one for those of Chou’s general PseAAC (122), including not only all the special modes of feature vectors for proteins but also the higher level feature vectors such as ‘Functional Domain’ mode, ‘Gene Ontology’ mode and ‘Sequential Evolution’ or ‘PSSM’ mode (122). Encouraged by the successes of using PseAAC to deal with protein/peptide sequences, the concept of PseKNC (Pseudo K-tuple Nucleotide Composition) (105) was developed for generating various feature vectors for DNA/RNA sequences (123–125) that have proved very useful as well. Particularly, recently a very powerful web server called ‘Pse-in-One’ (126) and its updated version ‘Pse-in-One 2.0’ (100) have been established that can be used to generate any desired feature vectors for protein/peptide and DNA/RNA sequences according to the need of users’ studies.

Since the reduced amino acids perform powerful ability, we firstly developed the flexible web server for generating pseudo K-tuple reduced amino acid composition (27). During the last 2 years, amounts of users’ feedback show that this online server must be updated by providing additional online services. Therefore, we update the online server, called RAACBook, which not only contained reduced amino acid analysis but also newly added the visualization report module, the comprehensive RAAC repository and the machine learning online tool. It performed more robust and powerful for simplifying protein complexity, providing feature files for prediction, training classification models and showing clearer conservative regions.

## Materials and Methods

### Data collection and curation

**Figure 2:**
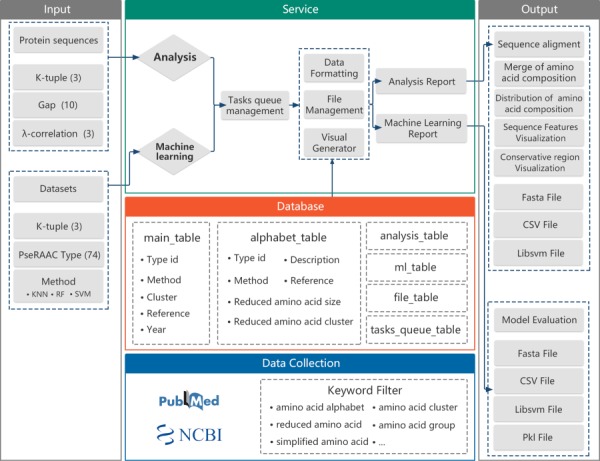
The framework of the RAACBook. Block diagrams showing the modules and functions of RAACBook. Input data are on the left, output data presented on the right. The data of the manually curated database is collected in PubMed by a keyword filter. Users can provide protein sequences in the webpage to generate reduced sequence vector files and visualizations. The users can also upload the protein sequence datasets as input to obtain the corresponding classifier model and evaluation.

The framework for the development of the RAACBook is described in Figure 2. The reduced amino acid alphabets of RAACBook were derived from over 1000 PubMed’s original literature, which were filtered by keywords as follows: ‘amino acid alphabet’, ‘reduced amino acid’, ‘amino acid cluster’, ‘amino acid group’, ‘simplified amino acid’ etc. Till 14 August 2019, 74 types of reduced amino acid alphabets were manually curated in RAACBook, which can generate 673 reduced amino acid descriptors for analyzing protein sequence (Supplementary Table 1). There are more than 40 clustering algorithms involved in this online repository, including BLOSUM matrix, maximum information gain, data mining and physico-chemical properties. In addition, the deep learning method has been also applied to the amino acid reduction (26). For better selecting desired alphabets, we developed a multilayer browser tool, which supports filtering of different keywords. The returned entries will link to the annotation information of RAAC, including description, reference and visualized clusters, which can be conveniently selected to reduced analysis.

### Implementation

The current version of the RAACBook database is constructed on MariaDB. The system is operated on Linux servers with 28 physical cores, which uses Apache as a proxy. The analysis engine and the visualization module are developed by Python.

After users upload protein sequences to the data management and set essential parameters, each job from the client can be submitted into the task queue with a unique Job ID. Job ID and parameters will be stored for the user to call again. As the function of the task queue management, if there are not enough computing resources available, the job is put on waiting schedule. For the user, job status is refreshed in the analysis report page. When the job is completed, the analysis report could be generated online.

As pointed out in (127) and demonstrated in a series of recent publications (67, 71, 72, 82, 128–144), user-friendly and publicly accessible web servers represent the future direction for developing practically more useful prediction methods and computational tools. Actually, many practically useful web servers have significantly increased the impacts of bioinformatics on medical science (54), driving medicinal chemistry into an unprecedented revolution (116).

## Results

### Overview of the RAACBook web service

To reduce complexity and understand topological estimation of protein sequences, natural protein sequences with the 20-letter amino acid alphabet can be commonly compressed to simplified alphabet based on some amino acid similar standards (98). RAACBook is an online repository of reduced amino acid alphabets. The current version contains RAAC database, reduction analysis, visualization and machine learning of protein classification (Figure 2). Firstly, The RAAC database provides a comprehensive resource of reduced amino acid alphabets. A multilayer browser is applied for filtering the desired RAAC from the database. Secondly, the analysis server can produce K-tuple reduced amino acid composition by defining three correlation parameters. The result provides reduced fasta files, csv and libsvm vector files for downloading. For different protein studies, we visualized alignment of sequences, mergence of amino acids, distribution of reduced amino acid composition, heat map of sequence features and logo of reduced sequence. Thirdly, the machine learning server enables users to build interesting classifier models, using different machine learning algorithms based on reduced sequence features, which can generate corresponding performance evaluation and downloadable file for each model.

### Reduction analysis of primary protein sequences

The major challenge in sequence-dependent inference is to extract the most efficient features and withdraw the information buried in primary protein sequences. To solve that, we developed the reduction analysis and the workflow is as below.

#### Data input and RAAC-type selection

**Figure 3:**
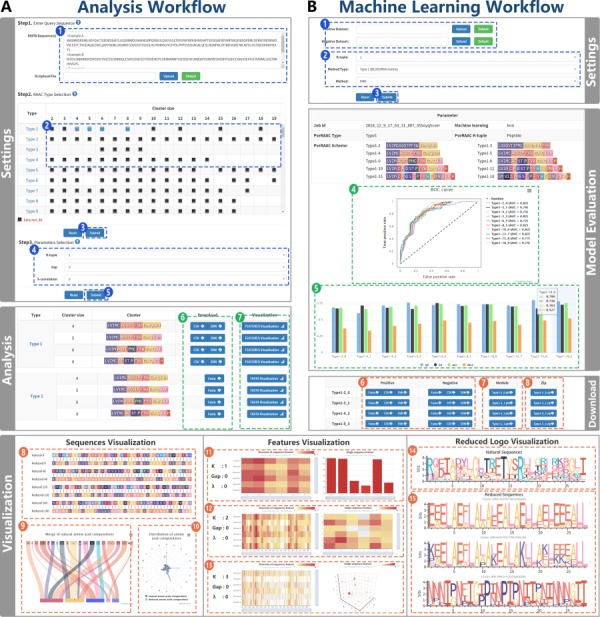
RAACBook analysis and machine learning workflow. Subfigure (**A**) The workflow shows the reduction analysis of natural amino acid sequence. Settings pane: After uploading primary sequences in fasta format (Step 1) and the alphabet types of interest were used as input (Step 2). If only these parameters are submitted, the server can generate reduced sequence files (Step 3). If the aim is to produce sequence feature files for machine learning, users need to select three parameters (Step 4) and submit (Step 5). Analysis panel: there are three files for download (Step 6). The reduced amino acid sequences are visualized by clicking ‘Visualization’ button (Step 7). Sequences visualization: three charts were exhibited: alignment between natural and reduced amino acid sequences (Step 8), mergence of natural amino acid composition (Step 9), and distribution of amino acid composition (Step 10). Features visualization (Steps 11–13): according to different reduced alphabets and parameters, service will generate the K-tuple reduced amino acid composition heat map of multiple sequences and the distribution of single reduced sequence peptides. Reduced logo visualization (Steps 14–15): the figure represents each amino acid information of each position in protein sequence based on the reduced alphabet. Subfigure (**B**) Machine learning workflow shows the acquisition of the classifier model by uploading datasets and setting parameters. Settings panel (Steps 1–3): K-tuple, the alphabet type and the machine learning algorithm are selected (Step 2), after uploading fasta files containing positive and negative datasets (Step 1). Subsequently, the machine learning service was executed (Step 3). Model evaluation: the chart of Sp, Sn, Acc, Mcc and the diagram of the ROC curve are generated (Steps 4 and 5), and the classifier model and vector files can be downloaded (Steps 6–8).

Users only need to upload the primary sequence with fasta format as input with three parameters and RAAC Type (Figure 3A, Step 1). The Types can be filtered in a two-dimensional selection box with the different alphabet types and cluster sizes, which are recorded in the RAACBook database (Figure 3A, Step 2).

#### Parameter selection

(i) K-tuple. The K-tuple value represents the number of peptide. For example, *K* = 1 means a monopeptide or amino acid, *K* = 2 represents a dipeptide, *K* = 3 represents a tripeptide, and so on. In a typical K-tuple analysis, one usually slides the window of width K amino acids along the protein by one residue at a time. For a protein with N amino acids, with *k* = 2, the dipeptide frequency be counted as follows, R1R2, R2R3, R3R4, etc. (Supplementary Figure 1A) (126,145). (ii) g-gap. The value of gap represents the inter-gap number between two nearest amino acid or K-tuple peptides along the protein. That is, the gap between each K-tuple peptide is represented. With *k* = 2, *g* = 1, the aim is to count the dipeptide frequency along the protein by skipping one residue in every slide as follows, R1R2, R3R4, R5R6, etc. (Supplementary Figure 1B) (126). (iii) λ-correlation. The λ-correlation of parameters, also called parallel correlation, which represents the gap number of each two adjacent amino acids in the K-tuple peptide interval. It is an integer greater than 2 and less than L-K, which reflects the protein sequence correlation between the nearest residue when K-tuple is determined (Supplementary Figure 1C). Taking *K* = 3, *λ* = 1, *g* = 2 as an example, the intra-gap number of within tripeptide interval is 1, and the number of skipping residue of tripeptide in each slide is 2. In the calculation process, the combination is R1R3R5, R4R6R8, R7R9R11 and so on (Supplementary Figure 1D).

The biological meaning of three parameters: it is well-known that the protein with specific domain codes endows sequence motifs with unique consensus, and the differential combination and arrangement determine the protein function. Here the K-tuple parameter was applied to calculate the total composition of n-peptide in the whole protein sequence. For example, when *K* = 3, the reduced tripeptide composition of protein sequence will be counted for 5TCD protein in Figure 1. It reflects the global composition of the secondary structure in 5TCD protein. The g-gap was introduced to compute the interspersed frequency of specific n-peptide with position bias. For example, ‘RR’ or ‘RRR’ can represent feature preference of alpha helix, which is usually scattered throughout the protein sequence. The *λ*-correlation was defined to extract feature information of function domain with low internal conservatism. Taking CXXC domains as example (Supplementary Figure 2), ‘X’ can be any amino acids, but the two external C residue is extremely conserved (146). This domain is ubiquitous in TET, DNMT and MBD protein. When *K* = 2, *λ* = 2, the feature preference of CXXC domain will be precisely captured by our feature extraction. Therefore, these three quantities will help researchers to obtain features with effective biological significance in meaningfully characterizing proteins.

#### Analysis report

The server will finally generate an analysis report of reduced amino acid, which consisted of parameter information, reduced feature and sequence files and visualization. The parameter information includes RAAC, K-tuple, g-gap, λ-correlation and job id. The download supports fasta, csv and libsvm vector files, and they are packaged as downloadable zip file (Figure 3A, Steps 6 and 7). Filtering to the desired feature vectors and sequence files is necessary for the prediction of protein three-dimensional structures or the construction of protein classification models. Therefore, the sequence reduction analysis includes the visualization to meet different studies, which consists of three parts: the sequence visualization, the feature visualization and the reduced logo visualization.

Sequence visualization includes three presentation methods (Figure 3A, Steps 8–10): firstly, in the alignment of the natural and reduced sequences, the colors used by the reduced amino acids are only a subset of those used by the natural ones. With such a color reduction in visualization, the primary structures of the proteins show clearer physical and chemical characteristics than the natural amino acid sequences. Also, the relevant protein complexity will be minimally degraded with nonessential information being suppressed, in some cases leading to more clearly display functionally conserved regions (147). Secondly, the matching relates the natural amino acids with the reduced ones. The lines represent association, and the width of a line is drawn in proportion to the frequency of involved amino acids in the reduction process. In general, some amino acids are more likely to reduce to certain amino acids, which show the preference in protein sequences. It plays a major role in structural biology, synthetic biology and new drug development. Thirdly, the frequency map of the natural and reduced amino acids is drawn.

Feature visualization (Figure 3A, Steps 11–13): according to different reduced alphabets and parameters, the server will generate the K-tuple reduced amino acid composition heat map of multiple sequences and the distribution of single reduced sequence peptides. Generally, the tool can provide the reference and helps users to find suitable vector files for their protein research.

Reduced logo visualization (Figure 3A, Steps 14 and 15): each logo is the representation of a multiple-protein sequence alignment, including natural sequence alignment and reduced sequence alignment. Each position of the sequence is a stack. Each stack is a collection of each amino acid frequency at this position (148). After the reduction, visualization provides a richer and clearer way to depict the sequences, such as binding sites and functional conserved regions.

### Machine learning of protein classification

As demonstrated by a series of recent publications (57, 60, 76–78, 82, 84, 88, 90–92, 128–132, 149–160) and summarized in two comprehensive review papers (122, 161), to develop a really useful predictor for a biological system, one needs to follow Chou’s five-step rule to go through the following five steps: (i) the valid benchmark datasets are firstly submitted as inputs for training the classifier model (Figure 3B, Step 1); (ii) the samples with an effective formulation can truly reflect their intrinsic correlation with the target to be predicted; (iii) the support vector machine (SVM), K-nearest neighbor (KNN) and random forest (RF) algorithm are introduced to operate the classifier; (iv) the 5-fold cross-validation is the default test for using to evaluate the anticipated accuracy of the classifier; and (v) establishment of a user-friendly web server for a classifier that is accessible to the public user. A new sequence-analyzing method or statistical predictor by observing the guidelines of Chou’s five-step rule has the following notable merits: (i) crystal clear in logic development, (ii) completely transparent in operation, (iii) easily to repeat the reported results by other investigators, (iv) with high potential in stimulating other sequence-analyzing methods and (v) very convenient to be used by the majority of experimental scientists.

The result of the machine learning server includes parameter information and model evaluation. The machine learning method, job ID, K-tuple and alphabet type are listed at the top of the report. The details of prediction for every alphabet are shown in the model evaluation section, including specificity (Sp), sensitivity (Sn), accuracy (Acc) and Mathew correlation coefficient (MCC) (Figure 3B, Step 5). The results of different alphabets are displayed in the receiver operating characteristic (ROC) curve, and the AUC value is given for reflecting the overall prediction ability (Figure 3B, Step 4). Finally, researchers can use the above indexes to select the appropriate classifier model or feature file to apply to their study. At the bottom of the report, users can conveniently download the fasta, csv, libsvm files and classifier models (Figure 3B, Steps 6–8).

### Applications

The reduced amino acid alphabets combined with machine leanings have been shown to have the ability for functional annotation of protein, such as iDPF-PseRAAAC (28), iHSP-PseRAAAC (149), Antimicrobial Peptide Scanner (26), Bastion6 (25) and iDNA-Prot|dis (162). A recent example of a collaborative focus within the RAACBook is the identification of secretory protein using RAAC, implemented as the ISP-PseRAAC. This is an online implementation of the SVM method, which can use protein sequences as input and provides prediction scores of secreted proteins. The valid benchmark datasets contain secretory proteins and non-secretory proteins of the malaria parasite. We use the SVM method to train the model by extracting three kinds of sequence feature including amino acid composition, dipeptide composition and tripeptide composition based on reduced amino acid sequence. Finally, based on dipeptide compositions of alphabet type 11 with cluster size 10, the prediction result of leave-one-out cross validation achieves 91.67% accuracy with 0.84 Mathew’s correlation coefficient, which demonstrates that the reduced alphabet has sufficient discriminatory power to predict protein function.

## New Features

The first version of RAACBook, PseKRAAC, was released as described by Zuo (27). The innovative features of the current version are as follows: (i) we increased the types of reduced amino acids alphabet from 16 to 74 and built a database for updating systematically the latest reduction type. These types contain nearly 700 clusters from literatures, each of which has a detailed method, description, reference, etc. (ii) The current web server rebuilt the user interface and background services, providing more friendly user interaction and a stronger system. Detailed tutorial and help are also supported throughout the use of the web server. (iii) The reduced analysis script was rewritten to improve efficiency, and analysis results were shown as a report, including a variety of downloadable files and images. In particular, we have added visualizations of reduced amino acid, sequence features and conservative region. (iv) We developed a machine learning tool to train online the classifier model and generated the evaluation report for users.

## Conclusion

The major challenge in protein sequence research, however, remains, for extracting precise information. The RAACs performed sufficient ability for decreasing protein complexity and withdrawing the conservative feature hidden in the noise signals that affect protein sequence researches. As new literature on amino acid reductions is published, we will update timely the database and server workflows to support the latest reduced alphabets and complicated studies. In short, RAACBook is a flexible and comprehensive web online platform where the hidden value of a large number of protein sequences can be explored by a wide range of users. With continuous user feedback and further enhancement, RAACBook has the potential to become an integral part of routine data of protein analysis for computational and experimental biologists.

## Supplementary Material

Supplementary_baz131
