# Supplementary material for: RAACBook: a web server of reduced amino acid alphabet for sequence-dependent inference by using Chou’s five-step rule
Source: Database (Oxford). 2019 Dec 5;2019:baz131. doi: 10.1093/database/baz131 (PMC6893003; doi:10.1093/database/baz131)
Supplement: Supplementary_baz131 [file supplementary_baz131.docx]

**Supplementary Data**

1. **Parameters**

**K-tuple**

The K-tuple value refers to the number of amino acid residues in the chain. K=1 means a peptide, K=2 represents a dipeptide, and so on. For each K-tuple of reduced amino acid cluster, the feature vector of the protein sequence contains RAAC^K^ dimensions. As the value of K increases, the vector dimension of RAAC^K^ will increase dramatically, causing the high-dimension disaster or overfitting, which reduces bias tolerance and cluster tolerance capacity. Therefore, the K value here is useful at a small time, so that we limit the K value to 3 to get a better reduction effect (Supplementary Figure 1A).

**Gap**

The value of gap is applied to the protein sequence that has been processed by K-tuple and represents the number of amino acid residues in the interval between each K-tuple peptide. That is, the g value reflects the sequence order information of all peptides, with the starting residues separated by g residues. For example, when g=1, the sequence is a peptide (K=1), then its former peptide contains R1R2, R3R4, R5R6, ... etc. (Supplementary Figure 1B).

**λ-correlation**

The λ-correlation of parameters, also called parallel correlation, is an integer and is less than K, where reflects the protein sequence correlation between nearest residue when k-tuple is determined. This is an integer representing the relevant layer, which reveals a protein sequence having a vector containing a RAAC^K^ component. For example, when K =3, λ=1, g=2, the combination is R1R3R5, R4R6R8, R7R9R11 and so on (Supplementary Figure 1C).


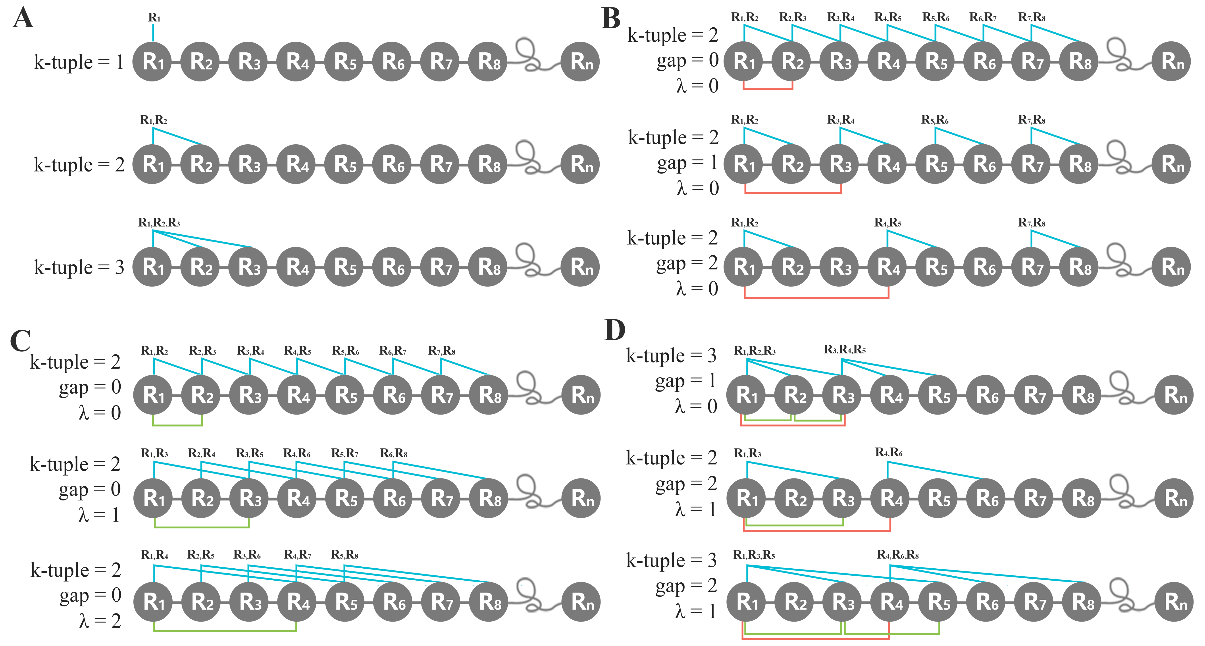


**Supplementary** **Figure 1.** Parameter of reduced amino acid


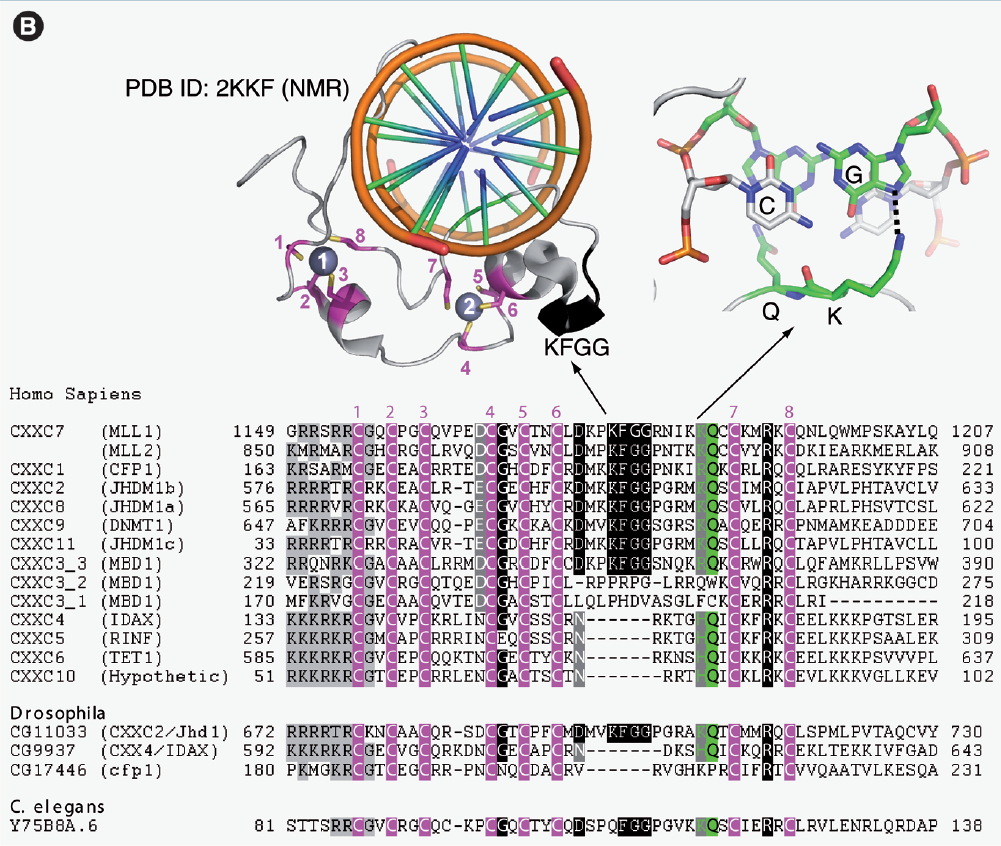


**Supplementary Figure 2 from reference** **(1).** CpG-interacting proteins

1. **Reduced amino acids alphabets**

Till 14 August, 2019, 74 types of reduced amino acid alphabets were manually curated in PseRAAC_Book, which can generate 673 reduced amino acid descriptors for analyzing protein sequence (2-41). In particular, we have added the latest amino acid reduction methods and clusters, such as deep learning, evolutionary algorithms and so on (Supplementary table1).

**Table 1.** Reduced amino acid alphabets.

| Type | Method description | Clusters | Dimension |
| --- | --- | --- | --- |
| 1 | BLOSUM50 | 2,3,4,5,6,8,10,12,15,18 | RAAC^K^ |
| 2 | BLOSUM40 | 2,3,4,5,6,7,8,9,10,11,12,13,14,15,16,17,18,19 | RAAC^K^ |
| 3 | Artificial immune system (AIS) | 6,6,6,7,7,8,8,8,8,9 | RAAC^K^ |
| 4 | Maximum information gain | 2,3,4,5,6,7,8,9,10,11,12,13,14 | RAAC^K^ |
| 5 | Secondary-structure | 2,3,4,5,6,7,8,9,10,11,12,13,15,16,17,18,19 | RAAC^K^ |
| 6 | Structure-Derived matrix（SDM） | 2,3,4,6,7,8,9,10,11,12,13,14,16,17,19 | RAAC^K^ |
| 7 | GONNET matrix | 2,3,4,5,6,7,8,9,10,11,12,13,14,15,17 | RAAC^K^ |
| 8 | Miyazawa Jernigan (MJ) matrix | 2,3,4,5,6,7,8,9,10,11,12,13,14,15,16 | RAAC^K^ |
| 9 | BLOSUM50 matrix | 2,3,4,5,6,7,8,9,10,11,12,13,14,15,16 | RAAC^K^ |
| 10 | Genetic algorithm | 5 | RAAC^K^ |
| 11 | Boltzmann relation/iteration | 2,3,4,5,6,7,8,9,10,14 | RAAC^K^ |
| 12 | Distance matrix | 2,4,7,12 | RAAC^K^ |
| 13 | Distance matrix | 2,3,5,8,12 | RAAC^K^ |
| 14 | Miyazawa Jernigan (MJ) matrix | 4,8 | RAAC^K^ |
| 15 | Protein blocks | 5,8,9,11,13 | RAAC^K^ |
| 16 | Hierarchical clustering | 2,3,5,7,8,11,14 | RAAC^K^ |
| 17 | Clustering analysis | 4,4,5,5,5,5,5,5,5,5,5,5,5,5,5,6 | RAAC^K^ |
| 18 | Physical-chemical property | 3,4,19 | RAAC^K^ |
| 19 | Maximized mutual information | 2,3,4,5,6,7,8,9,10,11,12,13,14,15,16,17,18,19 | RAAC^K^ |
| 20 | Variance maximization | 2,3,4,5,6,7,8,9,10,11,12,13,13,14,15,16,17,18,19 | RAAC^K^ |
| 21 | Extended compact genetic algorithm (ECGA) | 2,3,4,5 | RAAC^K^ |
| 22 | Extended compact genetic algorithm (ECGA) | 2,3,4 | RAAC^K^ |
| 23 | Extended compact genetic algorithm (ECGA) | 2,3,4,5,7,9,11 | RAAC^K^ |
| 24 | Physicochemical properties | 7,9,11 | RAAC^K^ |
| 25 | Extended compact genetic algorithm (ECGA) | 2,3,4,5 | RAAC^K^ |
| 26 | Extended compact genetic algorithm (ECGA) | 2,3,4 | RAAC^K^ |
| 27 | Extended compact genetic algorithm (ECGA) | 2,3,4,5 | RAAC^K^ |
| 28 | Miyazawa and Jernigan (MJ) matrix | 2,3,5,5 | RAAC^K^ |
| 29 | JTT rate matrix | 2,3,4,5,6,7,8,9,10,11,12,13,14,15,16,17,18,19 | RAAC^K^ |
| 30 | Grantham Distance Matrix | 2,3,4,5,5,7,8,9,10,11,12,13,14,15,16,17,18,19 | RAAC^K^ |
| 31 | Hierarchical clustering | 2,3,4,5,6,7,8,9,10,11,12,13,14,15,16,18,19 | RAAC^K^ |
| 32 | Hierarchical clustering | 2,3,4,5,6,7,8,9,10,11,12,13,14,15,16,17,18,19 | RAAC^K^ |
| 33 | Hierarchical clustering | 2,3,4,5,6,7,8,9,10,11,12,13,14,15,16,17,18,19 | RAAC^K^ |
| 34 | Unweighted pair-group method with arithmetic means (UPGMA) | 2,3,4,5,6,7,8,10,11,12,14,15,16,18,19 | RAAC^K^ |
| 35 | Dynamic clustering | 2,3,4,5,6,7,8,9,10,11,12,13,14,15,16,17,18,19 | RAAC^K^ |
| 36 | Information theory | 8 | RAAC^K^ |
| 37 | Physical-chemical property | 6 | RAAC^K^ |
| 38 | BLOSUM62 and Heuristic Monte Carlo (MC) method | 2,3,4,5,6,7,8,9,10,11,12,13,15,15,16,17,18,19 | RAAC^K^ |
| 39 | Chemistry properties | 3,4,4,8,10 | RAAC^K^ |
| 40 | Protein blocks | 4,8,9,10,10,13 | RAAC^K^ |
| 41 | Sequence alignments | 5,5,6,10,10,10,10,10,13,16 | RAAC^K^ |
| 42 | Structure alignments | 5,5,6,12,15,17 | RAAC^K^ |
| 43 | Contact potential | 5,5,5,5 | RAAC^K^ |
| 44 | Miyazawa Jernigan (MJ)matrix & BLOSUM50& BLOSUM62 | 13,14,19 | RAAC^K^ |
| 45 | Contact potential | 4,7.7 | RAAC^K^ |
| 46 | Miyazawa Jernigan (MJ)matrix and contact potential | 2,5,5 | RAAC^K^ |
| 47 | Physico-chdmical properties | 6 | RAAC^K^ |
| 48 | K-means | 8 | RAAC^K^ |
| 49 | BLOSUM62 and Heuristic Monte Carlo (MC) | 2,3,4,5,6,7,8,9,10,11,12,13,14,15,16,17,18,19 | RAAC^K^ |
| 50 | Dynamic Programming Alignments | 4,10 | RAAC^K^ |
| 51 | Unweighted variable group | 2,4,5,6,7,8,10,13,14,15,16,17,18,19 | RAAC^K^ |
| 52 | The Point Accepted Mutation (PAM) matrix | 2,3,4,5,6,7,8,9,10,11,12,13,14,15,16,17,18,19 | RAAC^K^ |
| 53 | Whelan and Goldman (WAG) matrix | 2,3,4,5,6,7,8,9,10,11,12,13,14,15,16,17,18,19 | RAAC^K^ |
| 54 | Physico-chdmical properties | 3,3,3,3,3,3,3 | RAAC^K^ |
| 55 | Hydrophobic and polar （HP）model | 4 | RAAC^K^ |
| 56 | Unweighted pair group method | 2,3,4,5,6,7,8,9,10,11,12,13,14,16,18,19 | RAAC^K^ |
| 57 | Unweighted pair group method | 2,3,4,5,6,7,8,9,10,11,12,13,14,15,16,17,18,19 | RAAC^K^ |
| 58 | Unweighted pair group method | 2,3,4,5,6,7,8,9,10,11,12,13,14,15,16,17,18,19 | RAAC^K^ |
| 59 | Unweighted pair group method | 2,3,4,5,6,7,8,9,10,11,12,13,14,15,16,17,18,19 | RAAC^K^ |
| 60 | Particle swarm optimization (PSO) | 5,6,8 | RAAC^K^ |
| 61 | Euclidean distance | 3,3,4 | RAAC^K^ |
| 62 | Physico-chdmical properties | 4 | RAAC^K^ |
| 63 | Miyazawa Jernigan (MJ)matrix | 2,3,4,5,6,7,8,9,10,11,12,13,14,15,16,18,19 | RAAC^K^ |
| 64 | Chemistry space | 5 | RAAC^K^ |
| 65 | Hierarchical clustering | 2,3,4,5,6,7,8,9,10 | RAAC^K^ |
| 66 | Hierarchical clustering | 2,3,5,6,7,10,11,13,15,16,18 | RAAC^K^ |
| 67 | Fuzzy clustering technique and matrices | 2,4,5,15,19 | RAAC^K^ |
| 68 | Fuzzy clustering technique and matrices | 2,3,5,7,14,19 | RAAC^K^ |
| 69 | Fuzzy clustering technique and matrices | 2,4,5,14,19 | RAAC^K^ |
| 70 | Fuzzy clustering technique and matrices | 5,7,8,19 | RAAC^K^ |
| 71 | Fuzzy clustering technique and matrices | 5,10,13,14,16,19 | RAAC^K^ |
| 72 | Fuzzy clustering technique and matrices | 2,3,5,7,14,19 | RAAC^K^ |
| 73 | Fuzzy clustering technique and matrices | 6,10,13,14,17,19 | RAAC^K^ |
| 74 | Fuzzy clustering technique and matrices | 5,11,15,18,19 | RAAC^K^ |

RAAC^K^: K-tuple of reduced amino acid cluster, for example, alphabet type 68, cluster = 7 and K-tuple=2, Dimension=7^2^

**References**

1. Hashimoto, H., Vertino, P.M., Cheng, X. (2010) Molecular coupling of DNA methylation and histone methylation. *Epigenomics*, **2**, 657-669.

2. Melo, F., Marti-Renom, M.A. (2006) Accuracy of sequence alignment and fold assessment using reduced amino acid alphabets. *Proteins*, **63**, 986-995.

3. Solis, A.D. (2015) Amino acid alphabet reduction preserves fold information contained in contact interactions in proteins. *Proteins*, **83**, 2198-2216.

4. Shepherd, S.J., Beggs, C.B., Jones, S. (2007) Amino acid partitioning using a Fiedler vector model. *Eur Biophys J*, **37**, 105-109.

5. Bacardit, J., Stout, M., Hirst, J.D.*, et al.* (2009) Automated alphabet reduction for protein datasets. *BMC Bioinformatics*, **10**, 6.

6. Smith, R.F., Smith, T.F. (1990) Automatic generation of primary sequence patterns from sets of related protein sequences. *Proc Natl Acad Sci U S A*, **87**, 118-122.

7. Yu, Z.G., Anh, V., Lau, K.S. (2004) Chaos game representation of protein sequences based on the detailed HP model and their multifractal and correlation analyses. *J Theor Biol*, **226**, 341-348.

8. Wang, J., Wang, W. (1999) A computational approach to simplifying the protein folding alphabet. *Nat Struct Biol*, **6**, 1033-1038.

9. Robson, B., Suzuki, E. (1976) Conformational properties of amino acid residues in globular proteins. *J Mol Biol*, **107**, 327-356.

10. Maiorov, V.N., Crippen, G.M. (1992) Contact potential that recognizes the correct folding of globular proteins. *J Mol Biol*, **227**, 876-888.

11. Veltri, D., Kamath, U., Shehu, A. (2018) Deep learning improves antimicrobial peptide recognition. *Bioinformatics*, **34**, 2740-2747.

12. Pape, S., Hoffgaard, F., Hamacher, K. (2010) Distance-dependent classification of amino acids by information theory. *Proteins*, **78**, 2322-2328.

13. Esteve, J.G., Falceto, F. (2004) A general clustering approach with application to the Miyazawa-Jernigan potentials for amino acids. *Proteins*, **55**, 999-1004.

14. Wrabl, J.O., Grishin, N.V. (2005) Grouping of amino acid types and extraction of amino acid properties from multiple sequence alignments using variance maximization. *Proteins*, **61**, 523-534.

15. Li, J., Wang, W. (2007) Grouping of amino acids and recognition of protein structurally conserved regions by reduced alphabets of amino acids. *Sci China C Life Sci*, **50**, 392-402.

16. Adamian, L., Liang, J. (2001) Helix-helix packing and interfacial pairwise interactions of residues in membrane proteins. *J Mol Biol*, **311**, 891-907.

17. Liu, B., Xu, J., Lan, X.*, et al.* (2014) iDNA-Prot|dis: identifying DNA-binding proteins by incorporating amino acid distance-pairs and reduced alphabet profile into the general pseudo amino acid composition. *PLoS One*, **9**, e106691.

18. Zhang, H., Kurgan, L. (2014) Improved prediction of residue flexibility by embedding optimized amino acid grouping into RSA-based linear models. *Amino Acids*, **46**, 2665-2680.

19. Jardin, C., Stefani, A.G., Eberhardt, M.*, et al.* (2013) An information-theoretic classification of amino acids for the assessment of interfaces in protein-protein docking. *J Mol Model*, **19**, 3901-3910.

20. Thomas, P.D., Dill, K.A. (1996) An iterative method for extracting energy-like quantities from protein structures. *Proc Natl Acad Sci U S A*, **93**, 11628-11633.

21. Stanfel, L.E. (1996) A new approach to clustering the amino acids. *J Theor Biol*, **183**, 195-205.

22. Kosiol, C., Goldman, N., Buttimore, N.H. (2004) A new criterion and method for amino acid classification. *J Theor Biol*, **228**, 97-106.

23. Susko, E., Roger, A.J. (2007) On reduced amino acid alphabets for phylogenetic inference. *Mol Biol Evol*, **24**, 2139-2150.

24. Solis, A.D., Rackovsky, S. (2000) Optimized representations and maximal information in proteins. *Proteins*, **38**, 149-164.

25. Davies, M.N., Secker, A., Freitas, A.A.*, et al.* (2008) Optimizing amino acid groupings for GPCR classification. *Bioinformatics*, **24**, 1980-1986.

26. Han, P., Zhang, X., Norton, R.S.*, et al.* (2006) Predicting disordered regions in proteins based on decision trees of reduced amino acid composition. *J Comput Biol*, **13**, 1723-1734.

27. Chen, Y.L., Li, Q.Z. (2007) Prediction of the subcellular location of apoptosis proteins. *J Theor Biol*, **245**, 775-783.

28. Li, Z.R., Lin, H.H., Han, L.Y.*, et al.* (2006) PROFEAT: a web server for computing structural and physicochemical features of proteins and peptides from amino acid sequence. *Nucleic Acids Res*, **34**, W32-37.

29. Etchebest, C., Benros, C., Bornot, A.*, et al.* (2007) A reduced amino acid alphabet for understanding and designing protein adaptation to mutation. *Eur Biophys J*, **36**, 1059-1069.

30. Weathers, E.A., Paulaitis, M.E., Woolf, T.B.*, et al.* (2004) Reduced amino acid alphabet is sufficient to accurately recognize intrinsically disordered protein. *FEBS Lett*, **576**, 348-352.

31. Li, T., Fan, K., Wang, J.*, et al.* (2003) Reduction of protein sequence complexity by residue grouping. *Protein Eng*, **16**, 323-330.

32. Sneath, P.H. (1966) Relations between chemical structure and biological activity in peptides. *J Theor Biol*, **12**, 157-195.

33. Li, X., Hu, C., Liang, J. (2003) Simplicial edge representation of protein structures and alpha contact potential with confidence measure. *Proteins*, **53**, 792-805.

34. Liu, X., Liu, D., Qi, J.*, et al.* (2002) Simplified amino acid alphabets based on deviation of conditional probability from random background. *Phys Rev E Stat Nonlin Soft Matter Phys*, **66**, 021906.

35. Murphy, L.R., Wallqvist, A., Levy, R.M. (2000) Simplified amino acid alphabets for protein fold recognition and implications for folding. *Protein Eng*, **13**, 149-152.

36. Cannata, N., Toppo, S., Romualdi, C.*, et al.* (2002) Simplifying amino acid alphabets by means of a branch and bound algorithm and substitution matrices. *Bioinformatics*, **18**, 1102-1108.

37. Atchley, W.R., Zhao, J., Fernandes, A.D.*, et al.* (2005) Solving the protein sequence metric problem. *Proc Natl Acad Sci U S A*, **102**, 6395-6400.

38. Prlic, A., Domingues, F.S., Sippl, M.J. (2000) Structure-derived substitution matrices for alignment of distantly related sequences. *Protein Eng*, **13**, 545-550.

39. Stephenson, J.D., Freeland, S.J. (2013) Unearthing the root of amino acid similarity. *J Mol Evol*, **77**, 159-169.

40. Mirny, L.A., Shakhnovich, E.I. (1999) Universally conserved positions in protein folds: reading evolutionary signals about stability, folding kinetics and function. *J Mol Biol*, **291**, 177-196.

41. Georgiou, D.N., Karakasidis, T.E., Nieto, J.J.*, et al.* (2009) Use of fuzzy clustering technique and matrices to classify amino acids and its impact to Chou's pseudo amino acid composition. *J Theor Biol*, **257**, 17-26.
